# The Molecular Architecture of Cell Adhesion: Dynamic Remodeling Revealed by Videonanoscopy

**DOI:** 10.3389/fcell.2016.00036

**Published:** 2016-05-04

**Authors:** Arnauld Sergé

**Affiliations:** Centre de Cancérologie de Marseille, Équipe “Interactions Leuco/Stromales”, Institut Paoli-Calmettes, Institut National de la Santé et de la Recherche Médicale U1068, Centre National de la Recherche Scientifique UMR7258, Aix-Marseille Université UM105Marseille, France

**Keywords:** diffusion, single molecule, super-resolution, nanoscopy, membrane dynamics, focal adhesion, synapse

## Abstract

The plasma membrane delimits the cell, which is the basic unit of living organisms, and is also a privileged site for cell communication with the environment. Cell adhesion can occur through cell-cell and cell-matrix contacts. Adhesion proteins such as integrins and cadherins also constitute receptors for inside-out and outside-in signaling within proteolipidic platforms. Adhesion molecule targeting and stabilization relies on specific features such as preferential segregation by the sub-membrane cytoskeleton meshwork and within membrane proteolipidic microdomains. This review presents an overview of the recent insights brought by the latest developments in microscopy, to unravel the molecular remodeling occurring at cell contacts. The dynamic aspect of cell adhesion was recently highlighted by super-resolution videomicroscopy, also named videonanoscopy. By circumventing the diffraction limit of light, nanoscopy has allowed the monitoring of molecular localization and behavior at the single-molecule level, on fixed and living cells. Accessing molecular-resolution details such as quantitatively monitoring components entering and leaving cell contacts by lateral diffusion and reversible association has revealed an unexpected plasticity. Adhesion structures can be highly specialized, such as focal adhesion in motile cells, as well as immune and neuronal synapses. Spatiotemporal reorganization of adhesion molecules, receptors, and adaptors directly relates to structure/function modulation. Assembly of these supramolecular complexes is continuously balanced by dynamic events, remodeling adhesions on various timescales, notably by molecular conformation switches, lateral diffusion within the membrane and endo/exocytosis. Pathological alterations in cell adhesion are involved in cancer evolution, through cancer stem cell interaction with stromal niches, growth, extravasation, and metastasis.

## Introduction

Cell junctions play a key role in the establishment and integrity of biological tissues, via protein–protein interactions at the cell surface. In multicellular animal organisms, mechanical integrity is ensured by diverse structures including *adherens* junctions, focal adhesions, desmosomes, and hemidesmosomes. Two other major functions of cell adhesion, which are not discussed here, concern epithelium and endothelium impermeability in-between cells by tight junctions, and direct communication between adjacent cells by gap junctions. In epithelia and endothelia, cells are connected, from apical to basal side, by the stratified structures of *zonula occludens* (tight junctions)*, zonula adherens* (adhesion belt)*, macula adherens* (desmosomes), gap junctions, and *basal lamina*. Present among virtually all cells, apart from cells of body fluids such as blood, lymph, or sperm, contacts are subject to physiological remodeling, notably during cell division and apoptosis. Transmembrane proteins generically named *cell adhesion molecules* (CAMs) interact either among adjacent cells or with the extracellular matrix (ECM) and are connected to the cytoskeleton by specific adaptors. The main CAM families encompass:

- Cadherins, in homophilic, calcium dependent cell–cell contacts.
- Integrins, in heterophilic, calcium/magnesium-dependent cell–matrix or cell–cell contacts.
- Selectins, in heterophilic (with sugar motifs), weak cell–cell contacts.
- Members of the immunoglobulin superfamily, in homo- or hetero-philic (with integrins), cell–cell contacts.

CAMs permit outside-in signaling, similar to membrane receptors, as well as inside-out, being susceptible to variations such as activation or aggregation by intracellular signals.

## Dynamic nanoscopy approaches: measures at high spatiotemporal resolution

**Figure 1:**
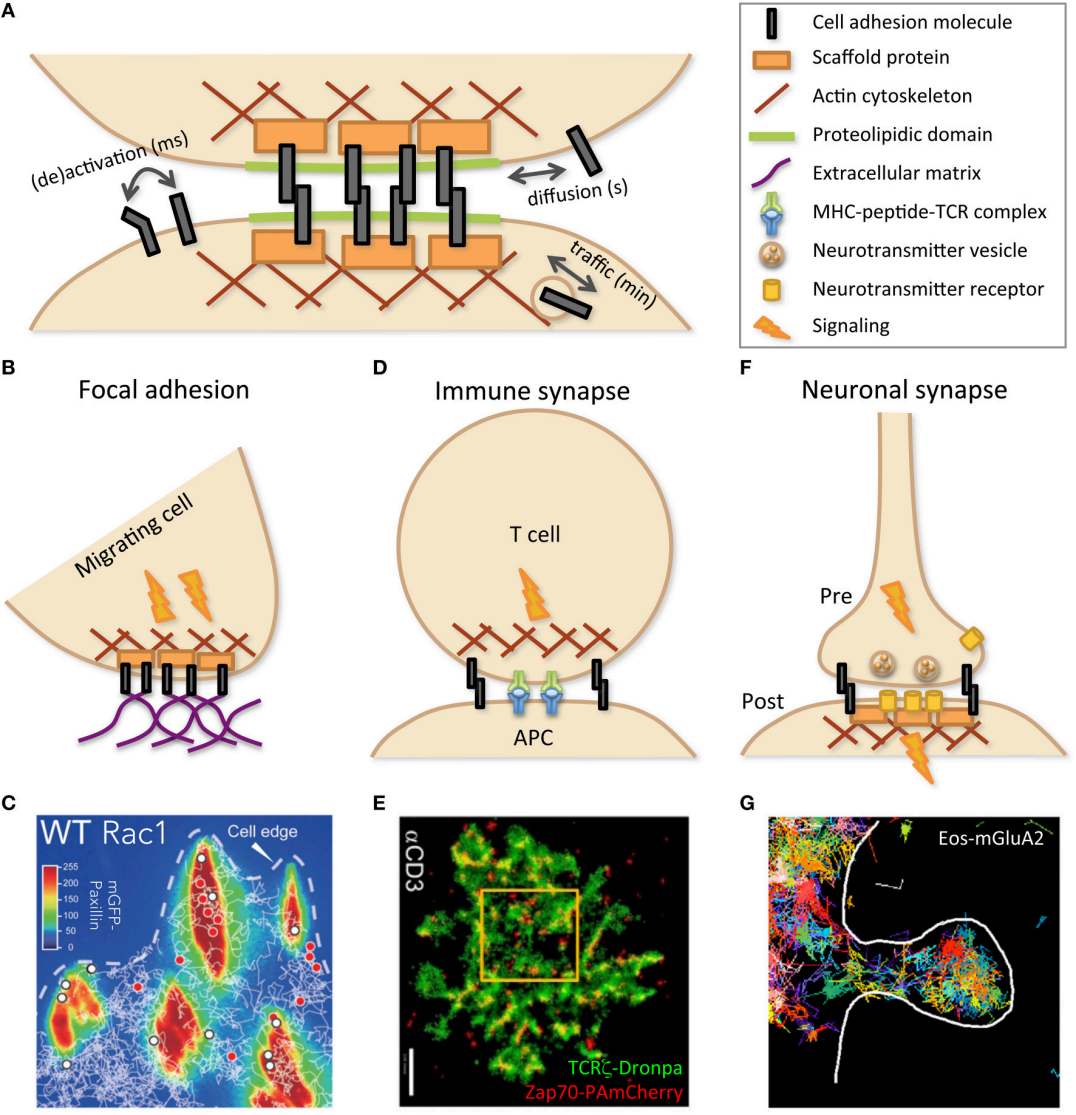
**Cell–cell adhesion is mediated by specific molecular structures. (A)** Schematic representation of the building blocks involved in cell–cell contacts. Dynamic evolution, as indicated by double arrows, may occur on various time scales, through changes in molecular conformation, such as activation, and localization, both within the membrane, by diffusion, and within the cell, by vesicular traffic. As depicted by cartoons **(B,D,F)** and illustrated by experimental data **(C,E,G)**, specialized cell contacts can be implicated in structures such as focal adhesion **(B,C)**, immune (between T cell and APC; **D,E**), and neuronal (between pre- and post-synaptic neurons) synapses **(F,G)**, dealing with specific dynamics in relation with their function. **(C)** Trajectories of wild-type (WT) Rac1, tagged with Halo-tetra-methyl-rhodamin, obtained by single-particle tracking (white lines) and superimposed on mGFP-Paxillin staining (false colors identifying FAs) reveal transient (red dots) or stable (white dots) immobilization within FAs. Reprinted from Shibata et al. (2013). **(E)** PALM imaging was performed with two molecules of the TCR complex, tagged with photoactivatable fluorescent proteins, TCRζ–Dronpa and ZAP-70-PAmCherry, in an E6.1 Jurkat cell on αCD3-coated coverslip. Nanoscopy of the immune synapse reveals TCR micro- and nano-clusters (green) with ZAP-70 sub-clusters (red) associated to activated TCR. Bar: 2 μm. Reprinted from Neve-Oz et al. (2015). **(G)** Trajectories of the tagged AMPA receptor Eos-GluA2 measured by sptPALM report transient organization in nanodomains within an excitatory dendritic spine (delimited by the white line) of a rat hippocampal neuron. Reprinted from Nair et al. (2013).

Cell contacts may be seen as static structures, through the classical representation provided by microscope images, usually obtained from fixed tissues. Yet, at the molecular scale, movements are essentially governed by thermal agitation, mostly leading to Brownian motion. This concept of dynamic molecular crowding applies to most cell constituents, including the plasma membrane, as described by the historical and still relevant fluid mosaic model (Singer and Nicolson, 1972). Molecular paths can be subjected to forces biasing Brownian motion and generating specific behaviors, like directed motion or permanent/transient immobilization (Sergé and Irla, 2013), particularly relevant for CAMs and their adaptors (Figure 1A). Hence cell contacts are permanently susceptible to evolve in composition and organization throughout their lifespan, from their establishment through remodeling and until disassembly.

Pioneer studies used methods such as Fluorescence Recovery After Photobleaching (FRAP), which was one of the first ways to measure the mobility of membrane components (Axelrod et al., 1976). Using antibodies or GFP as reporters, partial immobilization of CAMs such as integrins (Duband et al., 1988; Ballestrem et al., 2001) could be detected together with adhesion structures, during maturation and with associated partners such as the cytoskeleton and ECM. Other CAMs such as Junctional Adhesion Molecules (JAMs; Lamagna et al., 2005) and cadherins (Kusumi et al., 1993) were also studied by FRAP and by another technique that paved the way to single-molecule microscopy: Single-Particle Tracking (SPT) using antibodies coupled to latex or gold colloids of sub-diffraction size visualized by transmitted light. Documenting membrane events, like adhesion and endo/exocytosis, suffers from an intrinsically weak resolution along the optical axis (~500 nm). This can be circumvented by Total Internal Reflection Fluorescence microscopy (Axelrod, 1981). This configuration generates an evanescent field restricting illumination to ~100 nm above the coverslip, offering high axial resolution with reduced background and privileging visualization of the plasma membrane contacting the glass.

### Single-molecule microscopy

Advances in optical microscopy over the last few decades has allowed for the detection of a single fluorescent molecule with nanometer accuracy. Imaging a point source through a microscope is limited by diffraction, generating an Airy pattern, with a diameter of λ/2 NA (~200 nm), λ being the wavelength of light and NA the objective numerical aperture, as first determined by Abbe (1873). This pattern constitutes the *point-spread function* of the optical setup. The fluorophore localization, at the center of the Airy pattern, can be determined at high resolution providing sufficient signal-to-noise ratio, as predicted by Werner Heisenberg during the emergence of quantum theory (Heisenberg, 1927). Recent technological improvements in chemistry, optics and detectors have allowed for single-molecule detection in biological conditions. Seminal studies were first performed *in vitro*, with the pioneer observation of single enzyme activity, β-galactosidase (Rotman, 1961). An important breakthrough was later achieved by *fluorescence imaging with one-nanometer accuracy* to finely decipher myosin motion (Yildiz et al., 2003). Single-molecule observations were also reported in living cells, with pioneer works addressing transferrin (Byassee et al., 2000), epidermal growth factor (EGF; Sako et al., 2000), lipids (Schütz et al., 2000), calcium channel (Harms et al., 2001), and cadherin (Iino et al., 2001). We extended single-particle tracking by developing robust and efficient algorithms, named *multi-target tracing*, dedicated to the high probe density and low signal-to-noise ratio provoked by high acquisition rates (Sergé et al., 2008; Rouger et al., 2012). *Multi-target tracing* has been adapted to cell trajectories (Salles et al., 2013).

### Emergence of nanoscopy

The recent breakthrough of nanoscopy confirmed and further detailed previously unsuspected dynamic features of cell contacts. Single-molecule measurements require diluted enough dyes, separated on average by more than the Rayleigh criterion, 0.61 λ/NA. Two strategies were developed to surpass this limit. Stimulated-Emission-Depletion (STED; Hell and Wichmann, 1994; Hell, 2007) consists in generating an optical reduction of the *point-spread function* by using a depletion beam located around the excitation beam. Another approach has been provided by techniques such as Photo-Activated Localization Microscopy (PALM; Betzig et al., 2006; Hess et al., 2006) and Stochastic Optical Reconstruction Microscopy (STORM; Rust et al., 2006). Relying on a common scheme, these methods are collectively named Single-Molecule Localization Microscopy (SMLM). High-resolution images are built by iterative photoactivation of small subpopulations of dyes, sparse enough at each time-step to deliver single-molecule accuracy. Photoactivation/deactivation uses an appropriate strategy in each approach, i.e., switching photoactivatable proteins for PALM or controlling dye blinking for STORM. The image obtained by accumulating all localized molecules with SMLM can be termed pointillism, in reference to the painting technique. The obtained subwavelength resolution is only limited by signal-to-noise ratio and can be comparable to standard electron microscopy resolution (~50 nm). These imaging techniques allowed a technical and conceptual shift in spatial scales, from micro- to nano-scopy. In 2014, the Nobel Prize for Chemistry was awarded to Stephan Hell (Hell and Wichmann, 1994; Hell, 2007), Eric Betzig (Betzig, 1995; Betzig et al., 2006), and William Moerner (Moerner and Kador, 1989; Dickson et al., 1997) for these innovations. Nanoscopy have been extended to multicolor labeling (Bates et al., 2007; Meyer et al., 2008; Shroff et al., 2008), 3D (Huang et al., 2008; Punge et al., 2008; Vaziri et al., 2008; Shtengel et al., 2009), and living cells (Conley et al., 2008; Manley et al., 2008; Westphal et al., 2008), handling limits such as phototoxicity and artifacts putatively induced by tagging. Notably, nanoscopy revealed intense and unexpected dynamics of adhesion structures (Diez-Ahedo et al., 2009; Bakker et al., 2012; Rossier et al., 2012; Shibata et al., 2013; Ishibashi et al., 2015; Eich et al., 2016), revisiting the classical view of mostly static structures.

## Molecular organization of cell membranes

Several structures of the cell membrane play major roles in physiological functions through signaling and adhesion to neighbor cells and ECM. Generic features such as cytoskeleton meshwork, rafts, and protein complexes, which are subjected to thermal motion, can tailor the temporal evolution of membrane structures. Cell contacts benefit from proteolipidic domains to favor CAM aggregation, with the contribution of intracellular scaffolds and sub-membrane cytoskeleton. This leads to structures that are simultaneously elaborate and versatile, such as *focal adhesions* (FA; Rossier and Giannone, 2016), immune (Rossy et al., 2013), and neuronal (Maglione and Sigrist, 2013) synapses (Figure 1). Activation by fast and transient association of partners of a given signaling pathway, already localized in close proximity within narrow structures/domains, is a recurrent scheme to ensure fast and reliable signal transmission (Cebecauer et al., 2010).

### Submembrane skeleton fences and extracellular matrix

The actin cytoskeleton, in association with spectrin and transmembrane proteins, exhibits a gel organization constituting a meshwork located immediately beneath the plasma membrane. This meshwork not only mechanically reinforces and controls the shape of the membrane, but also constitutes barriers. Steric hindrance consequently constrains the diffusion of membrane components within domains, according to the *fences and pickets* model (Kusumi and Sako, 1996). Cytoskeleton meshes display sub-diffraction size, as imaged by electron microscopy and as evaluated from confined trajectories obtained by single-particle tracking. Instead of strict compartmentalization, dynamic evolution of the meshwork, allowing *hop* diffusion to adjacent domains, may lead to obstructed motion/anomalous diffusion (Fujiwara et al., 2002). Accordingly, on the extracellular side, the reticulated filaments of the ECM, tightly associated to the membrane glycocalyx, constitute a meshwork analogous to the cytoskeleton. Hence, ECM–cell contacts, notably via integrins, not only mechanically support tissues, but are also expected to obstruct or confine membrane component motion.

### Proteolipidic nanodomains/rafts

The *raft* hypothesis postulated that membrane lipids and proteins associate together according to their affinities, mostly emanating from their hydrophobicity and geometry (Simons and Ikonen, 1997). Indeed, the height of the intra-membrane part and a more or less cylindrical/conical shape, engendering a local curvature of the membrane, implicate an energetic cost. Cholesterol and saturated lipids such as sphingomyelin promote better packing within rafts. This leads to ordered and disordered phase separation co-existing within membranes, as first assessed by biochemistry. Rafts were initially proposed to contribute to protein sorting along the synthesis pathway, relying on the differential composition of the Golgi apparatus and other cellular compartments, with a key role attributed to cholesterol. They were also associated to several membrane features, including signaling platforms and adhesion structures. Rafts have been the focus of extensive research. Indeed, in contrast to the classic *floating island* metaphor, their putative sub-diffraction size and fast dynamics imply spatiotemporal characteristics just beneath the limit of most technological investigations.

### Other proteic domains and signaling complexes

Membrane components may also self-organize through attractive energetic potentials, typically generated by electrostatic and Van der Waals forces, even beyond rafts. Strikingly, in the retina, rhodopsin receptors display an almost crystalline packaging on the micrometric scale (Fotiadis et al., 2003). Proteic clusters also exist at sub-diffraction size (Daumas et al., 2003), like cytoskeleton meshes and rafts, with similar roles for integrating signaling partners within platforms (Douglass and Vale, 2005). Packing together effectors can be achieved through favorable energetic interactions, as well as by connections via scaffold proteins, reinforcing functional association with physical links. For instance, signaling crosstalk between integrin and major pathways, such as EGF, are physically reinforced via scaffolds like paxillin (Legate et al., 2006).

## Specialized cell contacts

Most cells are connected together to ensure proper mechanical and signaling coordination. Some contacts exhibit particularly complex dynamics and duration, as revealed by nanoscopy. We will focus on some emblematic contacts: FA, immune, and neuronal synapses (Figure 1). Migrating cells must establish strong though transient contacts along their path. Cell–cell contacts dedicated to information processing occur among immune and nervous cells and share the same term of *synapse*. Indeed, although the immune synapse is transient while the neuronal synapse may persist throughout life, they both contain similar features, including CAMs and signaling machinery, subjected to specific evolution over time (Dustin and Colman, 2002).

### Focal adhesion

FAs constitute a privileged site for mechanotransduction crosstalk between cells and ECM, mutually converting force sensing and signaling (Rossier and Giannone, 2016). FA nano-architecture was deciphered with 3D super-resolution using interferometric PALM. Axial position, usually poorly assessed, was determined at high resolution by analyzing the interference among the fluorescence collected by the two opposing objectives of a so-called 4π microscope (Shtengel et al., 2009). This allowed localizing FA components orthogonally to the plasma membrane, from integrins at the membrane, through adaptors such as paxillin and vinculin, to the actin cytoskeleton in the cytosol (Figure 1B). They notably established that talin, owing to its substantial size, crosses the whole structure (Kanchanawong et al., 2010). Comparable results were obtained for hemidesmosomes (Nahidiazar et al., 2015). Using videonanoscopy, the detailed dynamics and kinetics of integrins and their adapters were finely dissected, deciphering regulation by activation and association/dissociation to/from the cytoskeleton and ECM (Diez-Ahedo et al., 2009; Bakker et al., 2012; Rossier et al., 2012; Shibata et al., 2013; Ishibashi et al., 2015; Eich et al., 2016). This dynamic view of adhesions reveals an unsuspected plasticity in integrin number and residency time at FAs, modulated by pathophysiological conditions and extracellular signals to fine-tune ECM/cytoskeleton coupling (Figure 1C).

### Immune synapse

The immune synapse was initially conceptualized as the intimate contact established between a T cell and an antigen-presenting cell (Grakoui et al., 1999; Reichardt et al., 2009; Figure 1D). This was later extended to contacts implicating B cells and antigens (Harwood and Batista, 2010) or Natural Killer (NK) cells and target cells for delivery of lytic granules (Dustin and Long, 2010). In all cases, synapses contain specific receptors and are stabilized by CAMs. These molecules present a concentric organization, with receptors mostly at the central Supramolecular Activation Cluster (cSMAC) surrounded by CAMs such as LFA-1 at the periphery (pSMAC) and completed by distal elements with large extracellular domains (dSMAC). Immune synapse establishment (Klotzsch et al., 2015) and subsequent signaling (Salles et al., 2013) lead to antigen capture, lymphocyte activation, or target cell death. A precise choreography orchestrates co-receptors and partners associating/dissociating, as well as microtubules and their organizing center polarizing toward the cSMAC (Angus and Griffiths, 2013). STED nanoscopy revealed the intimate regulation of granule release by NK cells through the actin meshwork (Rak et al., 2011). Likewise, nanoscopy of TCR partners, Lat, and ZAP-70, documented spatiotemporal immune synapse organization, in coordination with signaling pathways, revealing patterning into micro- and nano-clusters that reorganize upon stimulation (Lillemeier et al., 2010; Sherman et al., 2011; Williamson et al., 2011; Neve-Oz et al., 2015; Figure 1E). Nanoscopy and single-particle tracking revealed actin reorganization upon lytic granules docking (Brown et al., 2011) and actin-mediated nano-clustering of CD1d in iNK T cells (Torreno-Pina et al., 2016). The relevance of dynamic studies performed *in vitro* that afford high resolution, like nanoscopy (Rossy et al., 2013), may be complemented by less resolved but more physiological *in vivo* measurements, notably intravital two-photon microscopy (Germain et al., 2012).

### Neuronal synapse

Like FAs and immune synapses, neuronal synapses also depict a complex sub-micrometric organization (Figure 1F). Single-molecule investigations have complemented neurophysiological approaches aimed at deciphering the molecular mechanisms that underlie pre- and post-synaptic plasticity. Synaptic receptors followed by single-particle tracking revealed that both inhibitory glycine and excitatory glutamate receptors reversibly aggregate together, through scaffold protein binding (Meier et al., 2001; Sergé et al., 2002). According to neuronal activity, synaptic efficiency can hence be modulated by receptor number at the post-synaptic density (Borgdorff and Choquet, 2002). However, due to their size, colloids were hampered to fully enter the synaptic cleft. This was circumvented by using smaller fluorescent labels (Dahan et al., 2003; Tardin et al., 2003). More recently, instead of monitoring a few labeled targets, nanoscopy provided a comprehensive view of the synapse at molecular resolution (Maglione and Sigrist, 2013). This further revealed dynamic spatial heterogeneity either pre- (Willig et al., 2006; Meyer et al., 2009; Ehmann et al., 2015), post- (Nair et al., 2013), or at the synaptic cleft (Perez de Arce et al., 2015). As for other cell contacts, lateral diffusion and vesicular trafficking constitute key solutions to modulate the spatiotemporal organization and function of synaptic components (Figure 1G).

## Cell contact evolution in cancer

**Figure 2:**
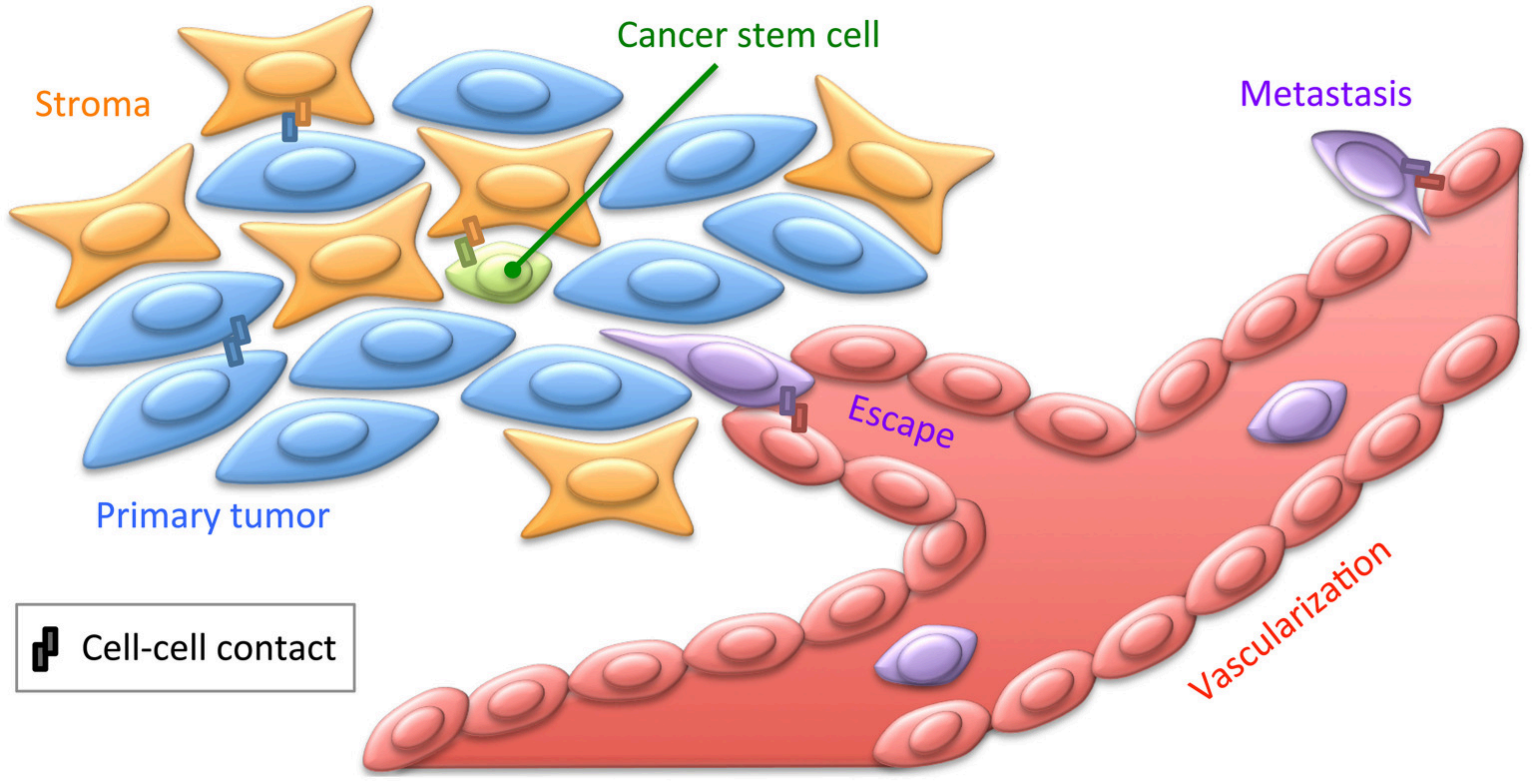
**Cell contacts in cancer**. Cancer cells engage contacts with themselves as well as with their surrounding stroma, including the ECM. Tumors are believed to contain cancer stem cells, engaging privileged contacts with the stroma allowing them not only to maintain quiescence and pluripotency, but also to putatively sustain resistance to chemotherapy. Cancer cells may also engage specific contacts with the tumor neovascularization. This may lead to cancer cell escape within the blood or lymph circulation by intravasation, and subsequently to distant metastasis by extravasation (Reymond et al., 2013). These various cellular interactions implicate a broad range of CAMs, such as cadherins, integrins, or JAMs, as well as ECM and soluble factors. Most of these cancer-specific contacts thus provide privileged strategies for immunotherapeutic treatments to target tumoral cells with monoclonal antibodies directed against integrins for instance (Scott et al., 2012).

Cancer primarily results from genetic alterations that lead to uncontrolled cell proliferation. Such control, critical for proper maintenance of cellularity within tissues, is achieved, at least in part, by signals emanating from contact inhibition. Epithelial–mesenchymal transition, physiologically required for embryonic development and wound healing, may also be dramatically hijacked in tumoral context, not necessarily for metastasis, but at least for chemo resistance (Fischer et al., 2015; Zheng et al., 2015). Hence, cell adhesion, in relation to signaling features such as rafts, is directly implicated in oncogenesis, often involving mutations leading to CAM up- and down-regulation (Eke and Cordes, 2015; Figure 2). NK cells constantly patrol organisms to detect and eliminate transformed cancer cells before massive tumor growth. As described above, super-resolution measures have improved our understanding of these cell-killing modalities (Dustin and Long, 2010; Brown et al., 2011). *In vitro* studies on fundamental cellular processes such as cell adhesion, immune interactions, as described above, as well as genome instability and cell division, are directly relevant for cancer research. Noteworthy, studies on integrin dynamics have been extended to cancer cells, revealing how the glycocalyx reinforces FAs and associated tumoral signaling (Paszek et al., 2014).

### Dissemination and metastasis by intra- and extra-vasation from vascularization

Tumor neovascularization is a major step in cancer progression. Upon hypoxia, the tumor and infiltrated leukocytes release growth factors stimulating angiogenic outgrowth of endothelial cells, sprouting from pre-existing neighboring blood vessels. CAMs, and in particular integrins, play crucial roles in tumor progression, and metastasis (Desgrosellier and Cheresh, 2010; Reymond et al., 2013). Leukocyte extravasation, essentially through endothelial tight junctions, is a mandatory step for tissue entrance. Transmigration requires complex interactions involving vascular CAMs such as vascular-endothelial-cadherin and members of the immunoglobulin superfamily, platelet-endothelial-CAM-1 and JAMs. These components of endothelial junctions are also directly involved in angiogenesis. Although, molecule and cell tracking share several analytic tools (Sergé and Irla, 2013) and apart from initial studies (Gonda et al., 2010), there is still a gap between nanoscopy**, mostly applied *in vitro* for molecular studies within cells, and intravital imaging, addressing cells within organisms. Indeed, intravital imaging adds several challenges, such as (i) managing animal breathing and heart beating and (ii) imaging at substantial depth within absorbing and scattering tissues, which will be challenging to reconcile with the mechanical stability and signal intensity required for nanoscopy. Data are thus essentially collected at cellular or subcellular scale, for specific molecules, although not at single-molecule resolution. Nevertheless, cancer evolution has been extensively documented regarding crucial steps such as dissemination and metastasis. Cells cultured within 3D matrix spheroids, thick tissue sections and dissected organs provide intermediate configurations from *in vitro* to *in vivo*, which are potentially better suited for nanoscopy (Ding et al., 2009; Cella Zanacchi et al., 2011). Further progress may allow *in vivo* investigations with nanometric resolution in the near future.

### Cancer stem cell interaction in niches

It is now established that cancers are not composed of homogenous clonal cells, but contain several cell types, differentiated to various extents. This includes cells exhibiting stemness properties, which are critical for two reasons: first, being quiescent, they escape most chemotherapies that target fast dividing cells as a classical hallmark of cancer, and second, they are susceptible to lead to relapse by differentiating and proliferating after treatment. One major point responsible for disparities among cancer cells is that they express distinct CAMs and thus differentially attach to each other and to the stromal microenvironment (Weidle et al., 2016; Figure 2). Membrane features such as rafts are directly implicated in stem cell retention in the stromal niche (Ratajczak and Adamiak, 2015). Molecular mechanisms allowing tumor cell localization within specialized microenvironments have been identified. Cancer relapse may arise from clonal re-emergence of cells kept quiescent in privileged microenvironments (Eppert et al., 2011). In the bone marrow, interactions between hematopoietic and stromal cells allow mutual transmission of signals involved in the development and homeostasis of both cell types (García-García et al., 2015). This crosstalk involves adhesion mechanisms, with a major impact on the development, maintenance, and proliferation of hematopoietic and stromal cells. Such interactions physiologically occur between JAM-C-expressing hematopoietic stem cells and JAM-B-expressing stromal cells (Arcangeli et al., 2011; De Grandis et al., 2016) and are extensively reorganized in leukemic context. Therefore, JAMs may provide a therapeutic target to block leukemic stem cell/stroma interactions responsible for resistance to treatment and relapse. Deciphering the *modus operandi* of JAMs in this process by nanoscopy could contribute in evaluating an adjuvant therapeutic potential for anti-JAM blocking antibodies to release leukemic cells from their niche.

## Concluding remarks

Adhesion is a common feature among nearly all cells within our organisms. CAMs are directly implicated in a broad range of physiopathological mechanisms related, for instance, to developmental defects, immunity and cancer. Increasing the resolution by one order of magnitude is a major breakthrough expected to deliver unsuspected structural and dynamic information on most cellular and cancerous processes, ranging from genomic to cell signaling mechanisms. This is also expected to aid in deciphering anti-tumoral mechanisms (Blom and Brismar, 2014), especially with respect to both spontaneous and therapeutic immunological responses. Upon examination at ever-increasing spatiotemporal resolution, subcellular structures reveal greater dynamics than previously assessed. In contrast from the static concept of CAMs and adaptors that would be definitively attached to FAs or synapses, nanoscopy offers a highly dynamic scheme of transient assemblies, emerging from stochastic motion and associations. Fast molecular reorganizations allow subtle cellular adaptions to environmental modifications. Photophysical performances, labeling specificity, and monovalency, with minimal artifacts induced by tagging, are important issues for nanoscopy, together with technological improvements in optics and sensors. Future directions will also include combining nanoscopy with complementary measures such as other imaging modalities, functional biochemical/electrical measures or single cell genomic/proteomic analyses. Microscopy modalities such as atomic-force microscopy and optical tweezers have also been considerably improved recently. Subcellular mechanical measurements allow us to include force as a new and important parameter when considering molecular interactions (Klotzsch et al., 2015). Understanding these subtle characteristics is of fundamental interest for the purpose of targeting and fine-tuning adhesion in pathologies such as cancer that profoundly implicate intercellular reorganization. Some processes, such as cancer dissemination and metastasis, intrinsically require considering a multicellular scale. Integrating super-resolution measures into whole organism or at least whole organ experiments will be another challenge. Pioneer work coupling two-photon with STED (Ding et al., 2009) or light sheet based planar illumination with SLSM (Cella Zanacchi et al., 2011), are promising steps toward intravital nanoscopy. Such experimental developments can be expected to find applications first in fundamental science before being potentially transferred to clinical use.

## Author contributions

The author confirms being the sole contributor of this work and approved it for publication.

## Funding

This work was supported by the ARC Foundation (PJA 20131200238) and institutional grants from INSERM and Aix-Marseille University.

### Conflict of interest statement

The author declares that the research was conducted in the absence of any commercial or financial relationships that could be construed as a potential conflict of interest.
